# PLGA-based drug delivery systems in treating bone tumors

**DOI:** 10.3389/fbioe.2023.1199343

**Published:** 2023-06-01

**Authors:** Enduo Qiu, Fei Liu

**Affiliations:** Department of Bone and Soft Tissue Tumor Surgery, Cancer Hospital of China Medical University, Liaoning Cancer Hospital & Institute, Shenyang, China

**Keywords:** bone tumors, PLGA, delivery systems, nanoparticles, scaffolds

## Abstract

Bone tumor has become a common disease that endangers human health. Surgical resection of bone tumors not only causes biomechanical defects of bone but also destroys the continuity and integrity of bone and cannot completely remove the local tumor cells. The remaining tumor cells in the lesion bring a hidden danger of local recurrence. To improve the chemotherapeutic effect and effectively clear tumor cells, traditional systemic chemotherapy often requires higher doses, and high doses of chemotherapeutic drugs inevitably cause a series of systemic toxic side effects, often intolerable to patients. PLGA-based drug delivery systems, such as nano delivery systems and scaffold-based local delivery systems, can help eliminate tumors and promote bone regeneration and therefore have more significant potential for application in bone tumor treatment. In this review, we summarize the research progress of PLGA nano drug delivery systems and PLGA scaffold-based local delivery systems in bone tumor treatment applications, expecting to provide a theoretical basis for developing novel bone tumor treatment strategies.

## Graphical Abstract

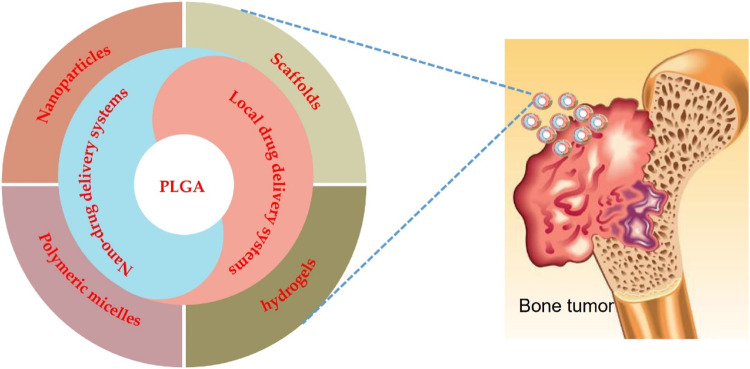

## 1 Introduction

**FIGURE 1:**
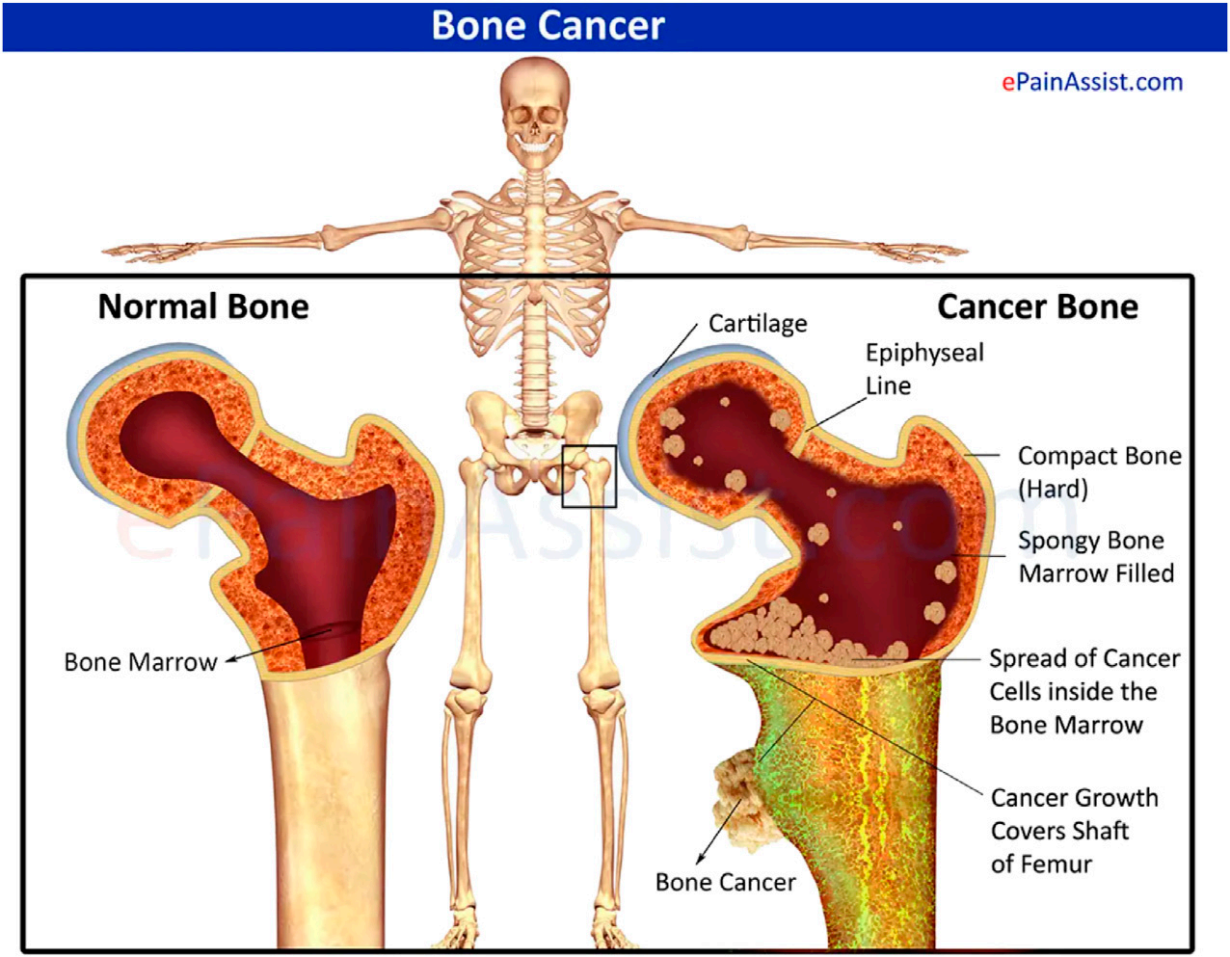
Schematic diagram of normal bone with cancerous bone. Reproduced with permission from [www.epainassist.com/bones/bone-cancer].

Bone tumors occur in the bones or their attached tissues (Figure 1), classified as benign or malignant (Choi and Ro, 2021), with the latter being more common in adults. Bone and joint pain, masses, and movement disorders are the main symptoms of bone tumors, especially malignant bone tumors, along with deformities, pathological fractures, and systemic symptoms such as insomnia, irritability, loss of appetite, depression, anemia, and cachexia (Bone Tumors, 2021). In line with WHO classifications, bone cancers can be categorized as primarily or secondarily malignant (Choi and Ro, 2021). Primary bone tumors are rare and originate from bone tissue, while secondary malignant bone tumors are those that metastasize to the bone from other tissues or organs in the body (Chowdhry et al., 2009). Despite the relatively low incidence, mortality from primary malignant bone tumors is very high. It has been reported that there were an estimated 24,000 cases and 17,200 deaths from primary bone cancer in China in 2014 (Xia et al., 2019), and 24,200 new cases and 17,900 deaths in 2015 (Xi et al., 2015).

Currently, the main treatments for bone tumors are surgery, chemotherapy (Table 1), and radiotherapy (Bădilă et al., 2021), among which the combination of preoperative and postoperative chemotherapy for limb-preserving surgery is the primary strategy (Ferguson and Turner, 2018). However, these treatments are associated with significant side effects and may not be effective in all cases; for example, surgical resection not only leads to large bone defects (Lu et al., 2019; Zhao et al., 2020a; Hayashi and Tsuchiya, 2022) but also fails to eradicate micrometastases, and the presence of residual tumor cells may increase the risk of disease metastasis and recurrence (Pantel et al., 2009; Massagué and Obenauf, 2016). Therefore, bone reconstruction and recurrence inhibition should be considered in the postoperative management of bone tumors. Administration of systemic chemotherapy/radiotherapy to patients is commonly used to suppress tumor recurrence; however, the liver, kidney, and digestive systems are stressed by high chemotherapy doses, which may lead to intense side effects (Oun et al., 2018; Schirrmacher, 2019). In addition, certain bone tumors, for example, osteosarcoma, are insensitive to radiation therapy and prone to chemoresistance (He et al., 2014; Kim and Kim, 2018). Hence, there is an urgent requirement to develop strategies to reduce systemic side effects and improve therapeutic efficacy, and there is a need for new and more effective treatments for bone tumors.

**TABLE 1:** The First-line therapy agents for osteosarcoma.

Chemotherapy agent	References
Cisplatin and doxorubicin	Kaneko et al. (2016), Zhang et al. (2018)
MAP (high-dose methotrexate, cisplatin, and doxorubicin)	Yu et al. (2019a), Zhang et al. (2020a)
Doxorubicin, cisplatin, ifosfamaide, and high-dose methotrexate	Sampson et al. (2013)
Ifosfamide, cisplatin, and epirubicin	Huang et al. (2015)

The elimination of tumor cells and the promotion of bone regeneration are the two critical issues in the treatment of bone tumors. Therefore, to address the clinical challenge of treating bone tumors and the demand for innovative approaches, researchers have focused on developing innovative strategies for bone tumor treatment, where biodegradable polymer-based targeted nano-drug delivery systems and local drug delivery systems have shown strong potential in terms of bone tumor therapeutic efficacy.

## 2 PLGA targeted nano-drug delivery systems

**FIGURE 2:**
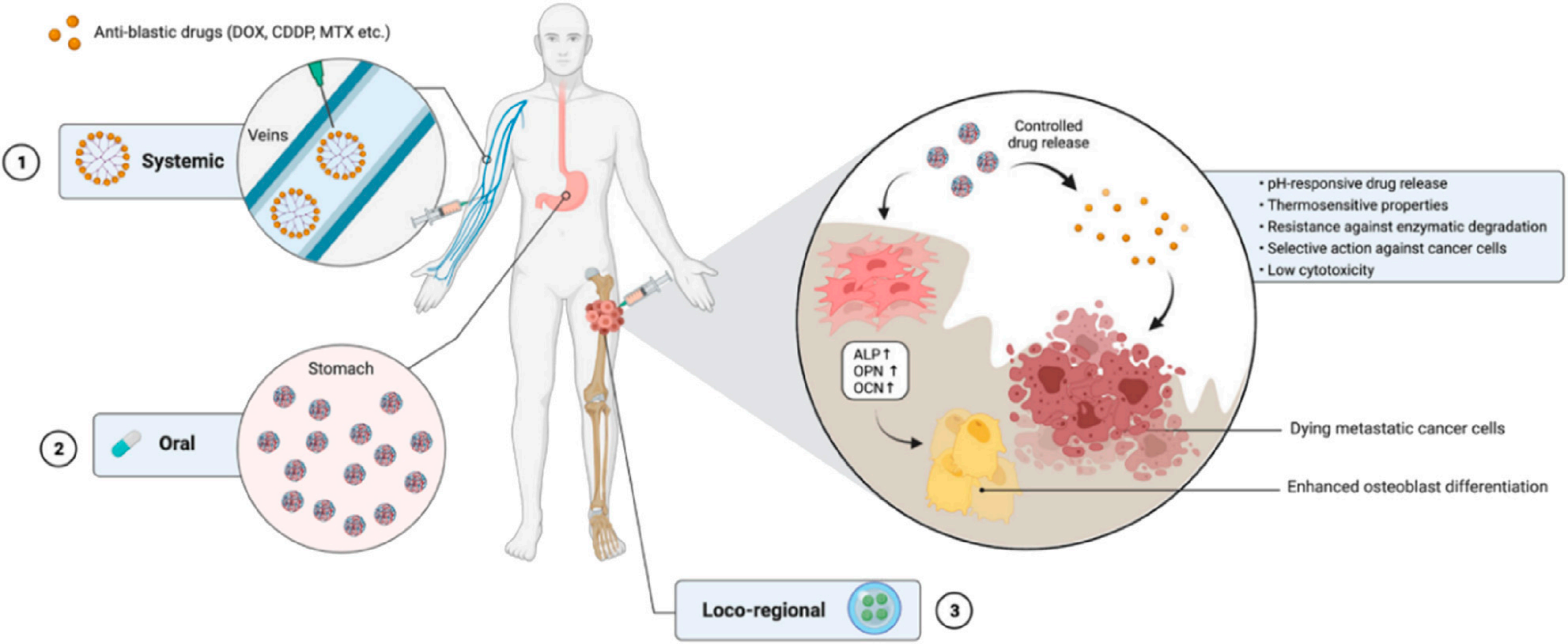
The use of nanocarrier-based drug delivery systems for the treatment of bone cancer. Reproduced with permission from Ambrosio L et al., ^©^ 2021 by the authors.

Much attention has been given to the potential of nanotechnology in drug delivery research, resulting in nanomedicine. The increasing interest in nanomedicine is due to the ability to improve cancer treatment by minimizing systemic toxicity and more efficacious targeted delivery of drugs (Shi et al., 2017; van der Meel et al., 2019; Wolfram and Ferrari, 2019; Zhang et al., 2020b; Germain et al., 2020; Irvine and Dane, 2020; de Lázaro and Mooney, 2021; Bhatia et al., 2022). The success of nanomedicine research will offer novel approaches for the ideal treatment of bone tumors (Figure 2). Targeting anti-cancer agents to the bone, either passively or actively, via nanocarriers is an attractive therapeutic approach for treating bone tumors, where the design of nanocarriers is vital to improve treatment efficacy and reduce the risk of adverse events (Ambrosio et al., 2021; Prasad et al., 2021).

### 2.1 PLGA nanoparticles

Among the biodegradable polymers developed for the formulation of polymeric nanoparticles, PLGA has attracted considerable attention and is widely used for its appealing characteristics, such as outstanding biocompatibility, favorable biodegradation, and FDA and EMA approval. PLGA-based delivery systems have demonstrated significant promise in treating bone tumors (Table 2).

**TABLE 2:** PLGA-based delivery systems investigated for treating bone tumors.

Anti-cancer drugs	Preparation methods	Particle size (nm)	Encapsulation efficiency (%)	Drug loading (%)	Treatment outcomes	Ref
Dox	solvent-displacement	245.400 ± 4.917	60.400 ± 0.600	—	Both DOX-loaded NP and free DXR reduced the Incidence of metastases in mice	Salerno et al. (2010)
Cabazitaxel	water-in-oil-in-water emulsion solvent evaporation	236.8 ± 1.19	55.87%	3.74%	Showed a significant reduction in tumor burden, reducing pain in the mouse tumor limb	Gdowski et al. (2017)
Bortezomib	solvent dispersion	195	24	0.74	Significantly enhanced survival and Decreased tumor burden in mice	Swami et al. (2014)
Dox	solvent diffusion	132 ± 9.5	73.53 ± 3.43	5.51	Showed prolonged blood circulation half-life, reduced liver uptake, and significantly higher retention of ZOL-tagged nps at the bone site with enhanced tumor retention	Chaudhari et al. (2012)
Gemcitabine and epirubicin	Solvent difusion or nano-precipitation	197 ± 7 (pH = 7.0)	89.8 ± 4.5	33.2 ± 4.4	Significant tumor (250%) regression was seen upon treatment with multiple drug-loaded zoledronic acid conjugated nanoparticle	Yuan et al. (2020)
Bisphosphonates	reverse microemulsion	178.1 ± 1.2	—	∼32%	Significantly inhibited tumor growth	Li et al. (2019a)
Ifosfamide	precipitation	124 ± 3.45	89 ± 1.95	20.15 ± 3.5	Exhibited remarkable *in vitro* anti-cancer activity	Chen et al. (2015)
Paclitaxel (PTX) and etoposide (ETP)	solvent evaporation	100 ± 3.68	92.5 ± 5.6	13.6 ± 2.8	Resulted in Enhanced cell cycle arrest and cell apoptosis	Wang et al. (2015)
Melatonin	emulsion–diffusion–evaporation	212.9 ± 65.6	17.3 ± 3.4	34.0 ± 4.3	Caused a toxic effect on the MG-63 cells	Altındal and Gümüşderelioğlu (2016)
Salinomycin	Emulsion diffusion evaporation	∼187 nm	60	—	Induced caspase-3 expression while Suppressing ß-catenin (wnt/ß-catenin pathway) and c-myc gene expressions in Osteosarcoma cancer cells	Irmak et al. (2020)
Ptx	nano-precipitation	132.73 ± 0.61	64.86 ± 0.17	4.24% ± 0.02	Suppressed tumor growth in tumor-bearing mice with minimal damage to normal tissues	Cai et al. (2022)
Ir780	water-in-oil-in-water (W/O/W) double-emulsion	236.8	67.8	3.25	Significantly induce HOS cells apoptosis and ferroptosis via excessive accumulation of reactive oxygen species	Wang et al. (2022a)

**FIGURE 3:**
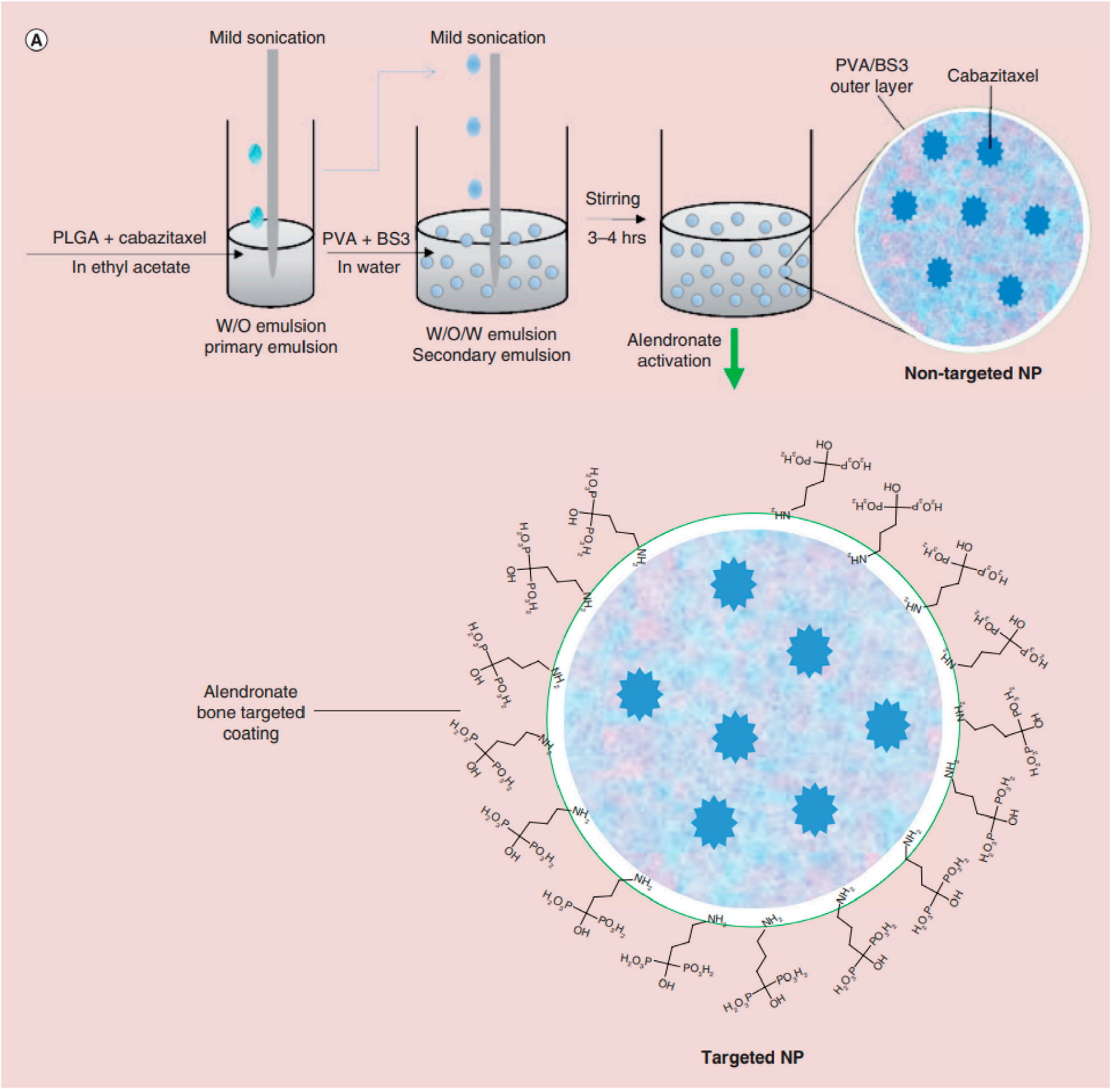
Schematic illustrating nanoparticle (NP) synthesis through water-in-oil-in-water double emulsion solvent evaporation technique followed by activation of nanoparticle with alendronate for bone targeting. Reproduced with permission from Gdowski A S et al., ^©^ 2017 by the authors.

Studies have shown that these systems provide controlled drug release, which can enhance their therapeutic efficacy while reducing the side effects associated with traditional chemotherapy. Additionally, PLGA-based systems have demonstrated high biocompatibility and biodegradability, which makes them ideal for long-term use for bone tissue engineering applications. Salerno et al. developed alendronate (ALN)-conjugated PLGA nanoparticles loaded with doxorubicin (DOX) for treating bone metastases from breast cancer (Salerno et al., 2010), and the obtained ALN-PLGA-DOX nanoparticles had good biocompatibility and the ability to target tumor-induced osteolytic sites (Cenni et al., 2008; Pignatello et al., 2009; Pignatello, 2011; Cenni et al., 2012; Thamake et al., 2012; Yin et al., 2016). *In vivo* studies showed that the ALN-PLGA-DOX nanoparticles could reduce the incidence of tumor metastasis and decrease the number of osteoclasts at tumor sites in mice. Gdowski et al. prepared ALN-conjugated PLGA nanoparticles loaded with cabazitaxel for enhanced drug delivery to the bone microenvironment (Figure 3). These bone-targeted PLGA-ALN nanoparticles significantly reduced tumor burden and pain in the tumor-bearing limbs of mice (Gdowski et al., 2017), representing a promising strategy for treating metastatic bone cancer. These results suggest that ALN-conjugated PLGA nanoparticles deserve further evaluation as practical for delivering anti-cancer drugs to bone tumors.

**FIGURE 4:**
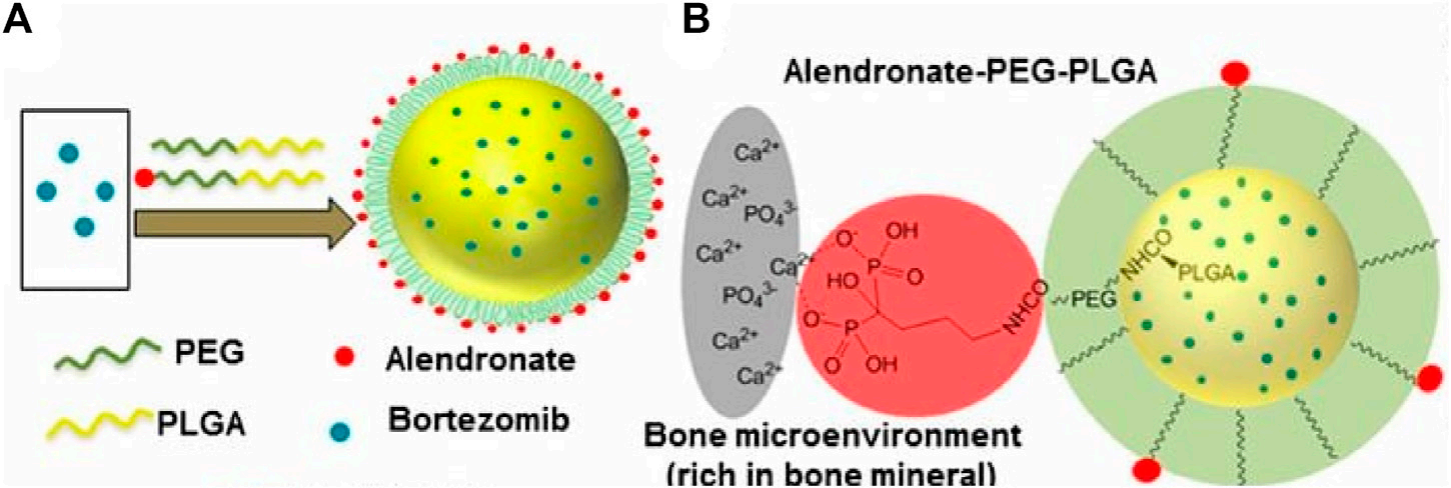
**(A)** Schematic illustration of alendronate-conjugated PEG-PLGA (Ald-PP) NPs synthesized by blending polymers (PLGA-b-PEG-Ald and PLGA-b-PEG) in varying ratios and encapsulating the drug bortezomib. **(B)** Schematic representation of the mechanism of affinity of Ald-PP NPs with bone mineral (gray, bone mineral; red, Ald; green, PEG; yellow, PLGA). Reproduced with permission from Swami A, S et al. Freely available online through the PNAS open access option.

**FIGURE 5:**
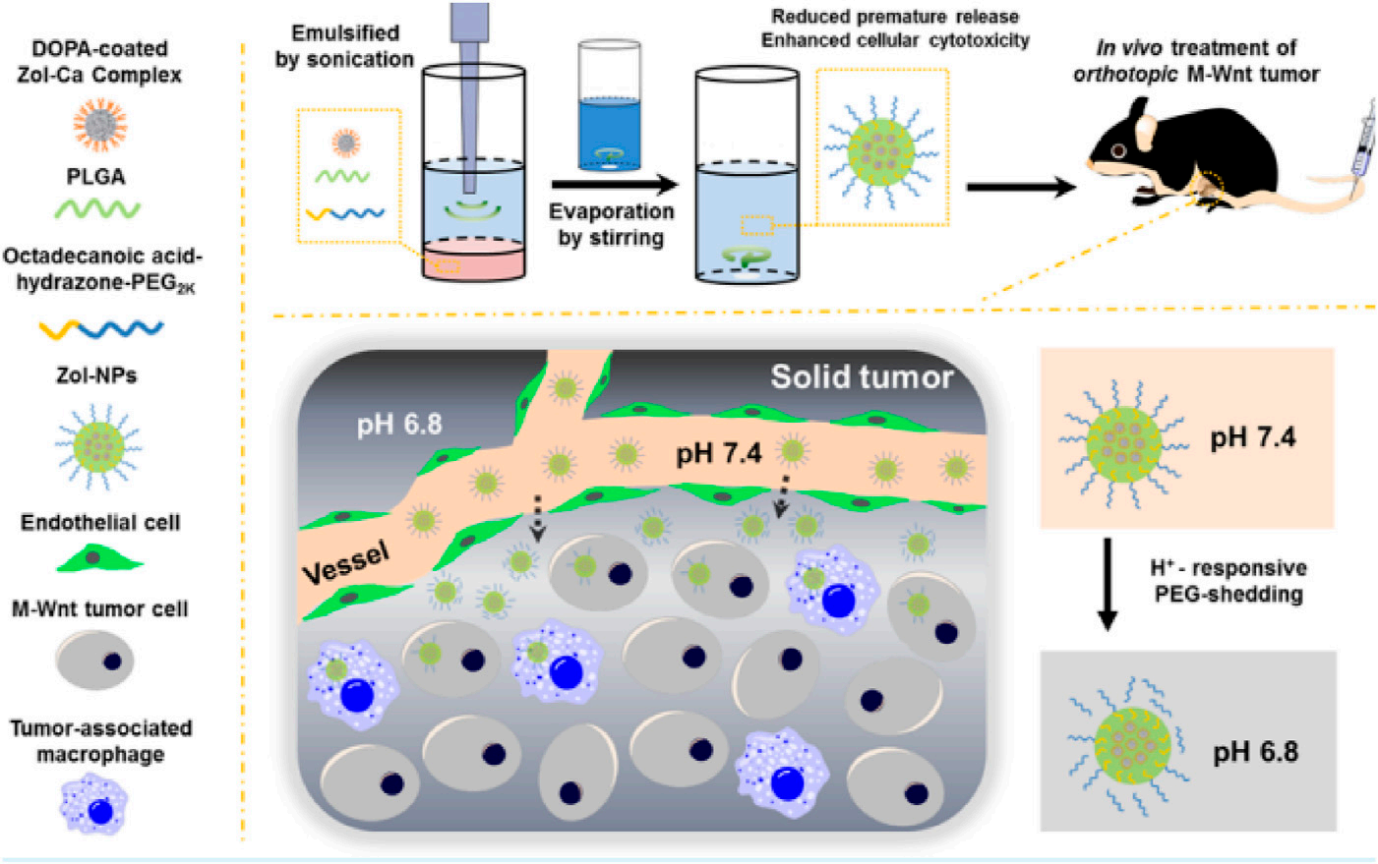
Schematic illustrations of zoledronic acid-containing nanoparticles with minimum premature release show enhanced activity against extraskeletal tumors. Reproduced with permission from Li X, S et al. Copyright ^©^ 2019, American Chemical Society.

Bortezomib shows significant anti-tumor activity in multiple myeloma. Swami et al. (Swami et al., 2014) found that bortezomib/PLGA nanoparticles inhibited myeloma growth in mice (Figure 4). Modifications of nanoparticle surface with PEG prevent clearance by the reticuloendothelial system. The Bortezomib-loaded ALN-PLGA-PEG nanoparticles exhibited good retentive, accumulative, and bone-homing properties. The zoledronate (ZOL)-conjugated PLGA-PEG-DOX nanoparticles also showed prolonged circulation half-life, reduced hepatic uptake, significantly longer tumor retention, and more effective prevention of multiple myeloma growth (Chaudhari et al., 2012). Similar observations of enhanced cellular uptake and increased tumor regression in mice (Li et al., 2019a; Yuan et al., 2020) were found for multiple drug-loaded PLGA-ZOL nanoparticles (Figure 5), demonstrating the conjugation of PLGA with a bisphosphonate with anti-tumor agents can be served as a potential treatment for osteosarcoma.

**FIGURE 6:**
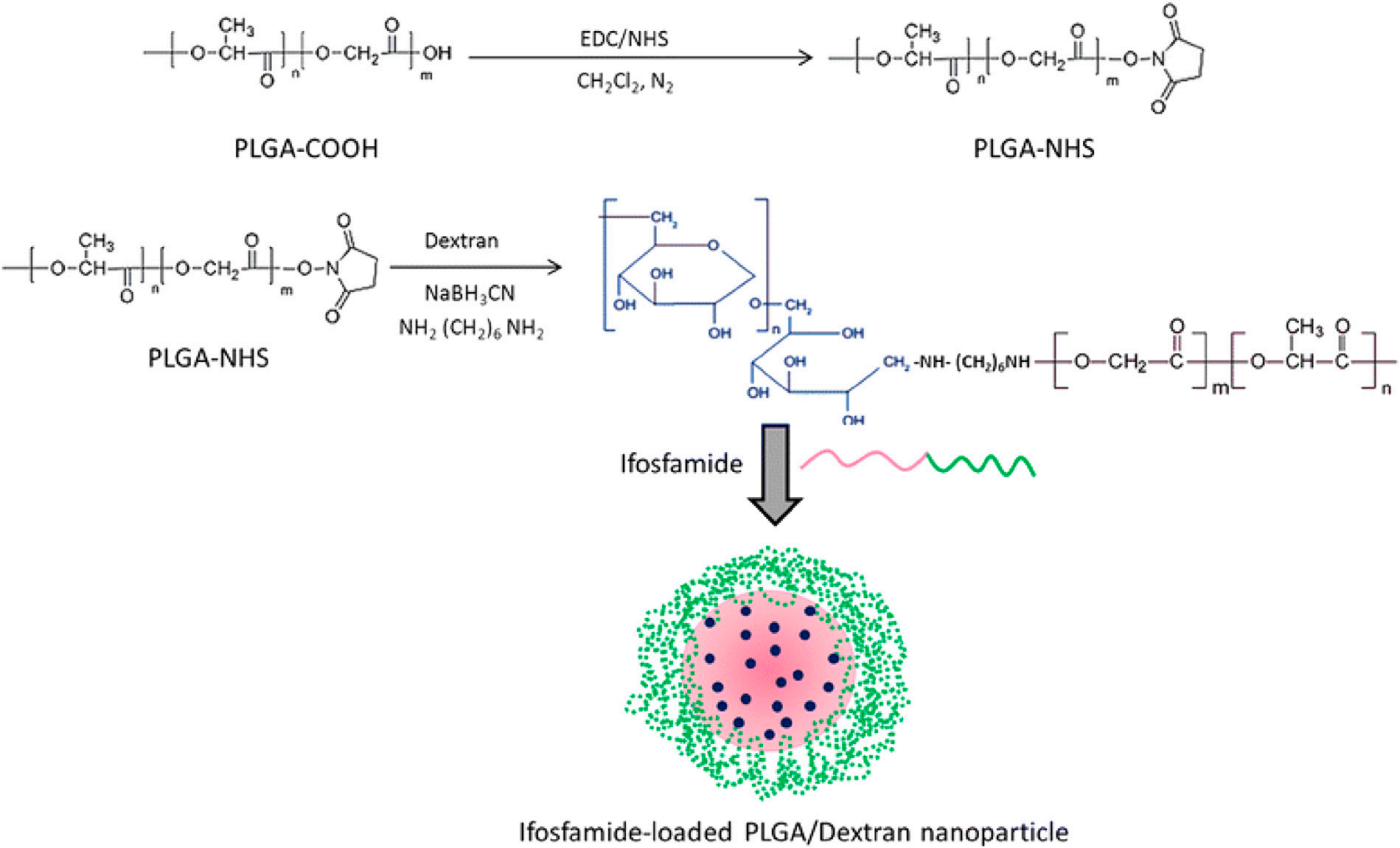
Schematic illustration of conjugation of PLGA polymer with the dextran block. Reproduced with permission from Chen B et al. Copyright ^©^ 2015, the authors.

Intravenous administration of ifosfamide (IFS) is indicated as the mainstay of treatment for osteosarcoma. Chen et al. enclosed ifosfamide in a conjugate of dextran and PLGA to evaluate its anti-cancer activity towards osteosarcoma tumor cells (Figure 6). The resulting particles showed a more significant promotion of cell death and apoptosis and superior *in vitro* anti-cancer activity compared to the free ifosfamide (Chen et al., 2015), suggesting that the PLGA nanoparticles obtained could be a possible therapeutic approach for treating osteosarcoma. Due to the complex microenvironment and drug resistance mechanisms of cancer, single-agent cancer therapy remains unsatisfactory (Liu, 2009; Bar-Zeev et al., 2017). Combination therapies are promising strategies to improve treatment efficacy and decrease adverse events (Xiao et al., 2017). The combined administration of more than one chemotherapeutic agent can produce synergistic suppression of cancer cells. Wang et al. encapsulated etoposide (ETP) and paclitaxel (PTX) into PLGA nanoparticles to investigate the synergistic effect of the combination regimen to promote apoptosis as a treatment for osteosarcoma (Wang et al., 2015). The resulting PLGA-PTX-ETP nanoparticles exhibited potent anti-cancer effects in MG63 and Saos-2 cancer cells in a time- and concentration-dependent manner. Co-administration of PTX and ETP led to the arrest of the cell cycle and enhanced cell apoptosis. The findings revealed that the combination significantly improved the treatment efficacy of the anti-cancer drugs. The combination of nanoparticles has a more significant inhibition effect, which will be beneficial for systemic cancer treatment. In conclusion, multi-drug PLGA nanoparticles will be a highly anticipated anti-cancer delivery system for treating osteosarcoma.

Melatonin has potent antioxidant, immunomodulatory, anti-proliferative, and tumor-suppressive properties (Moradkhani et al., 2020). Studies have shown that melatonin significantly inhibits tumor cell growth and can reduce chemotherapy drugs’ side effects (Bondy and Campbell, 2018; Gurunathan et al., 2021). Melatonin has a short half-life, which prolong its pharmacological effect. Altındal et al. used an emulsion-diffusion-evaporation method to encapsulate melatonin in PLGA nanoparticles, and MG-63 cells were significantly inhibited by melatonin/PLGA nanoparticles (Altındal and Gümüşderelioğlu, 2016). The results laid the foundation for studying melatonin/PLGA as an adjunct to conventional chemotherapy for osteosarcoma.

Salinomycin (SAL) has a highly selective anti-cancer activity. However, its low solubility in water reduces its therapeutic effect. To overcome this limitation, Irmak et al. encapsulated SAL in PLGA nanoparticles and evaluated the anti-cancer effect of the resulting delivery system. The results showed that PLGA/SAL nanoparticles could reduce proliferation and promote apoptosis of MG-63 cells more than SAL. The nanoparticles were also able to induce caspase-3 expression and inhibit that of β-catenin (Wnt/β-catenin pathway) and c-myc genes in osteosarcoma cancer cells, thus achieving a synergistic anti-cancer effect (Irmak et al., 2020). The findings showed that PLGA/SAL nanoparticles were faster and more effective than free SAL in eliminating osteosarcoma cells.

PLGA-carbon nanotube (CNT) conjugates provide binding targets for the caspase-3 (CP3) to form a CNT-PLGA-CP3 coupling that can effectively transduce cells and inhibit cell proliferation for up to 1 week at doses down to 0.05 μg/mL. These results are critical in demonstrating the ability of gene delivery using PLGA-functionalized CNTs for cell fate regulation (Cheng et al., 2013). The PLGA nanoparticles loaded with other anti-cancer agents, such as sclareol (Cosco et al., 2019), castalin (Zhang et al., 2022), and simvastatin (Venkatesan et al., 2019; Jin et al., 2021a), also present potent anti-cancer activity, indicating that PLGA nanoparticle delivery systems have promising potential for treating bone cancers.

**FIGURE 7:**
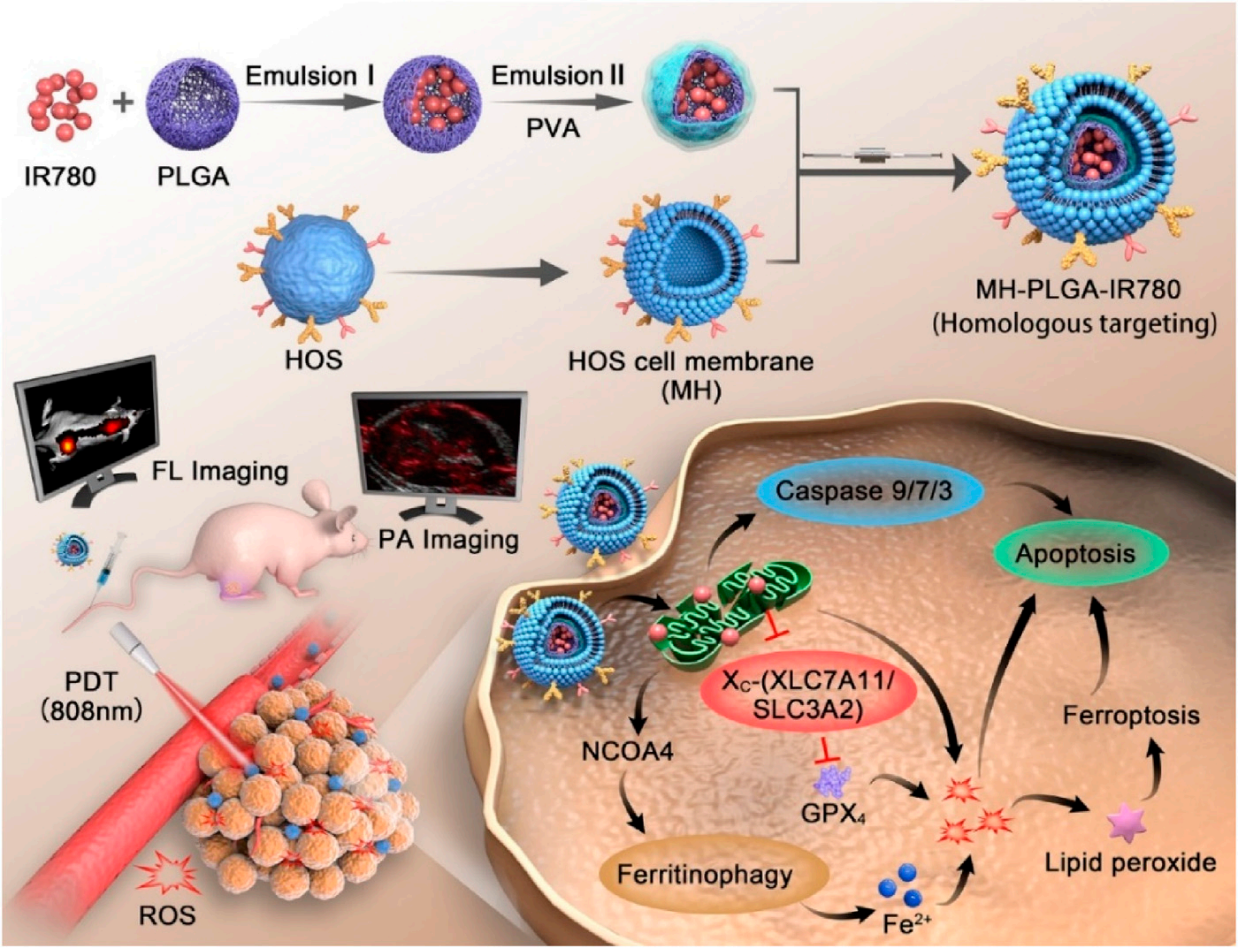
Schematic illustration of the construction of the MH-PLGA-IR780 NPs and the specific killing mechanism of the targeted theranostic nanoplatforms-mediated PDT approach. Reproduced with permission from Wang Y et al. Copyright ^©^ 2022, The Author(s).

Although PLGA-based nanoparticles have been intensively explored for targeted cancer therapeutics because they promise to improve and prolong the efficacy of conventional anti-cancer drugs with fewer adverse effects, they suffer from limitations, including clearance by the reticuloendothelial system and insufficient penetration capacity to tumor cells (Zhang et al., 2016; Wang et al., 2020a). Membrane extracted from tumor cells could be employed for coating nanoparticles to achieve homologous targeting (Sun et al., 2016). Furthermore, nanoparticles coated with tumor membranes exhibited high conjugative capacity and specific uptake into homologous tumor cells, leading to lower levels of immune-clearance after routine administration (Harris et al., 2019). To take advantage of homologous targeting and minimized immunological clearance, Cai et al. prepared paclitaxel (PTX) loaded PLGA nanoparticles encapsulated in osteosarcoma cell membranes and macrophage membranes for the targeted delivery of chemotherapeutic agents to osteosarcoma (Cai et al., 2022). *In vitro* studies demonstrated that PTX-PLGA nanoparticles enhanced the PTX uptake by the tumor cells and caused apoptosis in the osteosarcomas. They showed excellent tumor-targeted activity and significant tumor growth inhibition and were less toxic than free PTX. The research offers a targeted delivery approach for osteosarcoma therapy. The human osteosarcoma cell membrane-coated PLGA nanoparticles loaded with IR780 significantly improved endocytosis *in vitro* and tumor accumulation *in vivo* and induced considerably apoptosis and iron phagocytosis in HOS cells by excessive accumulation of ROS (Figure 7). At the same time, the PLGA-IR780-loaded nanoparticles-guided PDT also significantly inhibited tumor growth *in vivo* (Wang et al., 2022a).

### 2.2 PLGA-based polymeric micelles

Polymeric micelles are nanometer-sized (5–100 nm) colloidal particles that readily self-assemble from amphiphilic polymers (Jones and Leroux, 1999; Movassaghian et al., 2015). Over the past 20 years, polymeric micelles have received considerable research focus in drug delivery, with particular emphasis on their possible uses in the main areas of delivering drugs, including drug solubilization, controlled drug release, and drug targeting (Croy and Kwon, 2006; Miyata et al., 2011; Ahmad et al., 2014). Polymer micelles have gained attention as a novel approach to treating and diagnosing cancer due to various advances compared to conventional drug delivery. These include the following key advantages, 1) improved drug solubility: many anti-cancer drugs have poor solubility, which can limit their effectiveness. Polymeric micelles can enhance drug solubility, allowing for improved drug delivery and efficacy; 2) selective targeting: polymeric micelles can be engineered to target specific cells or tissues, including cancer cells. This allows for more precise drug delivery and reduces the risk of off-target effects; 3) reduced toxicity: Polymeric micelles can help reduce the toxicity by minimizing their exposure to healthy cells and tissues, reducing the risk of side effects and improving patient outcomes; 4) enhanced circulation time: polymeric micelles can increase the circulation time of drugs in the body, allowing for sustained drug release and improved therapeutic efficacy; 5) enhanced accumulation of anti-cancer drugs in the tumor: Their enhanced permeability and retention (EPR) effects allow them to accumulate in the tumor microenvironment. Overall, the advantages of polymeric micelles make them promising for treating cancers (Biswas et al., 2016; Yu et al., 2019b; Majumder et al., 2020; Ghosh and Biswas, 2021; Hari et al., 2023). One clinically successful micelle formulation is Genexol, which is paclitaxel encapsulated in a polylactic acid polymer micelle and was approved in Korea in 2007 for treating breast, lung, and ovarian cancer (Egusquiaguirre et al., 2012; Cabral and Kataoka, 2014; Deshmukh et al., 2017; Kar et al., 2020; Norouzi and Hardy, 2021).

PLGA-based polymer micelles have demonstrated significant promise in treating bone cancers (Table 3). Research studies have shown that these micelles can effectively deliver anti-cancer drugs to tumor locations and increase the effectiveness of the drugs. In addition, PLGA-based polymeric micelles have also been found to enhance drug solubility and bioavailability, thereby reducing the side effects associated with chemotherapy. For example, PEG–PLGA micelles were used to encapsulate the gallium (III)-diflunisal complex for treating osteosarcoma, and the results showed that the resulting nanoparticle formulations were 5,645 times more potent against osteosarcoma than doxorubicin and cisplatin. The nanoparticle formulation also induced nuclear damage to DNA, downregulation of cyclooxygenase-2, and caspase-driven apoptosis (Passeri et al., 2023).

**TABLE 3:** PLGA-based polymer micelles investigated for treating bone tumors.

Anti-cancer drugs	Preparation method	Particle size (nm)	Encapsulation efficiency (%)	Drug loading (%)	Treatment outcomes	Ref
Gallium (III)-diflunisal complex 1	Nanoprecipitation	95.5	24.1 ± 0.7	4.8 ± 0.1	Exhibits up to 5645-fold greater potency towards oscs than doxorubicin and cisplatin	Passeri et al. (2023)
DOX and ALN	Solvent diffusion	202 ± 4.2	83.7 ± 1.23	71.89	Displayed superior cytotoxicity in MG-63 cells	Liu et al. (2016)
Dextran	W/O/W Double emulsion	∼180	>90	28.5	Exhibited significant apoptosis of mg63 cancer cells, significantly higher g2/m phase arrest in mg63 cells, showed a most significant anti-tumor activity with maximum tumor growth inhibition	Liu et al. (2015)
Mithramycin	Emulsion Solvent evaporation	210–267	87	—	Efficiently inhibits the signaling mediated by the pro-oncogenic factor SP1	EstupiñánRendueles et al. (2021)

Liu developed chitosan (CS)-conjugated PLGA micelles loaded with docetaxel (DOC) and alendronate (ALN) to increase the therapeutic efficiency in osteosarcoma cells. CS-conjugated PLGA with dual-drug-loaded (DTX and ALN) micelles exhibited typical time-dependent cellular uptake and also showed higher cytotoxicity in MG-63 cells in comparison to blank micelles, which were found to be safe and biocompatible. The findings suggested that the combined loading of DTX and ALN into micelles enhances the therapeutic efficacy of the formulation for osteosarcoma therapy (Liu et al., 2016).

Cisplatin (CDDP) is a potent anti-cancer drug commonly used in the treatment of osteosarcoma, but the efficacy of CDDP is limited by severe undesirable side effects such as renal toxicity and neurotoxicity. The formulation of CDDP in polymer micelles is anticipated to reduce the adverse related impact while improving efficacy. Liu et al. encapsulated CDDP in ALN-PLGA micelles (PLD) conjugated with dextran (DX) to enhance the specificity of the delivered system for bone tumor cells and improve the efficacy of osteosarcoma treatment. *In vitro* cellular cytotoxicity tests showed that PLD/CDDP micelles had outstanding anti-cancer activity and exhibited substantial cellular uptake via an endocytic-mediated mechanism. In comparison with free CDDP, PLD/CDDP had a marked apoptosis effect on MG63 cancer cells. The most important thing, PLD/CDDP demonstrated the most pronounced anti-tumor efficacy and the highest tumor inhibition, suggesting excellent anti-cancer potential in osteosarcoma. Taken together, the PLD/CDDP micelles significantly improve the anti-cancer activity of CDDP in osteosarcoma cells, and PLD-containing CDDP formulations may represent a most promising and efficacious therapeutic approach for the control of osteosarcoma (Liu et al., 2015).

Miller et al. developed PEG-PLGA micelles for the co-delivery of PTX and ALN, which synergistically target bone metastases from breast cancer. *In vitro* results have shown that the PTX-PLGA-ALN micelles have almost identical cytotoxic as well as anti-angiogenic features to those of the free drugs. Furthermore, in a model of mCherry-infected tibia cancer, PTX-PLGA-ALN micelles achieved superior efficacy and safety compared to free PTX. The results showed that the selective accumulation of the PTX-PLGA-ALN micelles in the tumors resulted in a higher inhibition of tumor growth than the controls (Miller et al., 2013).

Mithramycin A (MTM), a natural chrysophanol polyketide, has been used to treat several types of cancer. Despite its efficacy, serious adverse effects have hindered its use in clinical practice at the concentrations necessary to achieve a beneficial therapeutic outcome. Estupiñán et al. provided MTM-loaded PLGA micelles for efficient nano-delivery of MTM. These MTM nano delivery systems mimic the potent anti-tumor efficacy of free MTM in both liposarcoma and chondrosarcoma models. As with free MTM, nanocarrier-delivered MTM also effectively suppressed SP1-mediated signaling. This suggests they may provide a safer delivery option for MTM that could be investigated for clinical use in the future (EstupiñánRendueles et al., 2021).

These studies suggest that PLGA-based polymeric micelles have great potential for treating bone cancers and could provide a promising alternative to traditional chemotherapy. At the same time, further exploration is necessary to fully understand the potential of these micelles in the treatment of bone cancer. The initial results are promising and suggest they may be a viable treatment option.

## 3 PLGA-based local drug delivery systems

Local drug delivery systems offer the opportunity to advance the efficiency and acceptability of cancer therapy by maximizing drug delivery to the target site. Polymeric hydrogels loaded with anti-cancer agents can be delivered close to the tumor of interest and generate adequate concentrations of the drug locally (Cirillo et al., 2019; Fan et al., 2019; Kesharwani et al., 2021; Xiao et al., 2021; Yu et al., 2021). Because the drug is administrated locally rather than intravenously, serious side effects due to high drug levels would be minimized (Wei et al., 2017; Zheng et al., 2017; Darge et al., 2019; Zhou et al., 2019; Rafael et al., 2021). Injectable and biodegradable hydrogels have attracted substantial attention in the biomedicine field because they allow for more precise implantation into hard-to-reach tissue sites and site-specific delivery, as well as their injectability and biodegradability (Li et al., 2012; Lee et al., 2019; Mallick et al., 2020; An et al., 2021; Zhou et al., 2021). Among them, The FDA-approved amphiphilic triblock copolymers PLGA-PEG-PLGA have been employed to prepare thermosensitive hydrogel formulations and received much interest for their medical application potential due to their excellent biological compatibility and biodegradation (Gao et al., 2011; López-Cano et al., 2021; Ghandforoushan et al., 2022; Heine et al., 2022; Lin et al., 2022; Yuan et al., 2022).

### 3.1 PLGA-based hydrogels for local drug delivery

**FIGURE 8:**
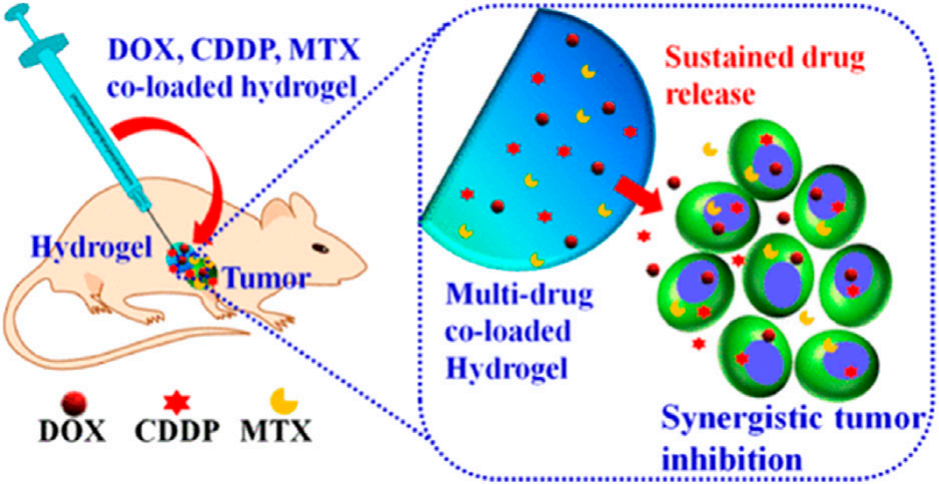
Schematic illustration of the co-delivery of doxorubicin, cisplatin, and methotrexate by thermosensitive hydrogels for enhanced osteosarcoma treatment. Reproduced with permission from Ma H et al. Copyright ^©^ 2015, American Chemical Society.

PLGA-based hydrogels have shown great promise in the therapy of bone tumors. These hydrogels can be loaded with drugs or other therapeutic agents and implanted into the bone, where they slowly release the medicine over time. This approach allows accurate and selective drug delivery directly to the cancer cells with minimal adverse effects on surrounding normal tissues (Jin et al., 2021b; Liao et al., 2021). The FDA-approved PLGA-PEG-PLGA triblock copolymer is a candidate for the local delivery vehicle to bone tumors (Marques and Kumar, 2022). Ma et al. developed a novel strategy for osteosarcoma therapy using PLGA-PEG-PLGA hydrogel to release a combination of drugs, such as MTX, CDDP, and DOX (Figure 8). The results showed that the hydrogels co-loaded with multiple drugs displayed cytotoxicity that was synergistic towards the tumor cells *in vitro*. Following injection into Saos-2 osteosarcoma xenografts, drug-loaded hydrogels provided the most potent tumor suppression *in vivo* for 16 days (Ma et al., 2015). Furthermore, systemic toxicity was reduced, and no significant injury. Thus, local co-administration via a PLGA-PEG-PLGA hydrogel may be a viable therapy to enhance the treatment efficacy of osteosarcoma. The authors also used PLGA-PEG-PLGA hydrogel for local delivery of PLK1shRNA/polyether-modified polyethylene glycol (PEI-Lys) complex and DOX for osteosarcoma therapy. The resulting drug delivery system showed significant and synergistic effects in inducing osteosarcoma cell apoptosis when cultured with Saos-2 and MG63 osteosarcoma cells. Following subcutaneous injection of the drug delivery system adjacent to Saos-2 osteosarcoma in nude mice, the hydrogels demonstrated more excellent anti-tumor activity *in vivo* (Ma et al., 2014). In particular, the combined *in vivo* therapy resulted in almost total inhibition of tumor proliferation for 16 days without significant organ damage, suggesting that co-delivery of PLK1 shRNA and DOX by PLGA-PEG-PLGA hydrogels might offer the opportunity for effective therapy of osteosarcoma in the clinic.

To develop a more effective combination therapy for treating osteosarcoma, Yang et al. used PLGA-PEG-PLGA as a vehicle to deliver DOX and β-cyclodextrin-curcumin (CD-CUR) to the target tumor in a controlled manner. The co-delivery system demonstrated superior anti-tumor efficacy and a greater capacity to induce apoptosis. Similarly, local PLGA-PEG-PLGA combination treatments exhibited a greater anti-tumor efficiency *in vivo* as compared to free DOX + CD-CUR or single-agent strategies (Yang et al., 2020). In another research, a PLGA-PEG-PLGA hydrogel loaded with DOX and CDDP was utilized for local combination chemotherapy of osteosarcoma. The resulting hydrogel was degradable and biocompatible. *In vitro* cell viability assays showed that the hydrogels co-loaded with DOX and CDDP exhibited synergistic anti-proliferative effects as well as high tumor growth inhibition efficiency. The *in vivo* experimental results indicated that the PLGA-PEG-PLGA hydrogel for local combination chemotherapy promoted increased tumor necrosis and enhanced the modulation of the expression of apoptosis-related genes (Si et al., 2022), indicating synergistic anti-tumor effectiveness *in vivo* with low systemic toxicity. These results indicated that persistent local co-delivery of DOX and CDDP via PLGA-PEG-PLGA hydrogels could be a promising approach to effectively treating osteosarcoma in the clinic.

Although PLGA-PEG-PLGA hydrogels have shown considerable therapeutic promise in local cancer treatment, the maximum tolerated dose (MTD) of these strategies is still unclear. To address this issue, Yang et al. employed PLGA-PEG-PLGA hydrogels loaded with DOX to evaluate the MTD of DOX in the local treatment of osteosarcoma (Yang et al., 2018). The hydrogels exhibited favorable injectable and biodegradable properties *in vivo*, with significantly prolonged drug residence time at the tumor location. The results showed that the local administration of DOX at 5.0 mg/kg could not suppress tumor-sustained growth or extend the survival duration, while the local administration of DOX at 30 mg/kg showed intense activity in suppressing tumor growth but also caused severe weight loss. At the same time, the local administration of DOX at 15 mg/kg showed markedly higher anti-tumor potency and extended average survival time as compared to free DOX (15 mg/kg). The improved MTD and reduced systemic toxicity of DOX administered with hydrogels will offer a potential therapy for osteosarcoma.

### 3.2 PLGA-based scaffolds for local drug delivery

Recently, local DDS based on bone scaffolds has attracted increasing interest. Drug-loaded scaffolds can generate high local drug concentrations to eradicate residual tumor cells. This approach promises to prevent tumor recurrence and reduce systemic adverse events more effectively than intravenous drug delivery. The right biomaterial can both treat tumors and promote bone regeneration. Compared to natural polymers, synthetic polymers offer adjustable properties for bone scaffolds, allowing the control of molecular weight or functional group ratios to modulate mechanical strength, degradation, and other properties. Among synthetic polymers, PLGA is the most commonly used to fabricate porous scaffolds due to its controlled biodegradability, limited toxicity, and potential capabilities. PLGA-based scaffolds have been developed for the localized delivery system to treat bone tumors and regenerate bone (Sarigol-Calamak and Hascicek, 2018; Wang et al., 2022b).

**FIGURE 9:**
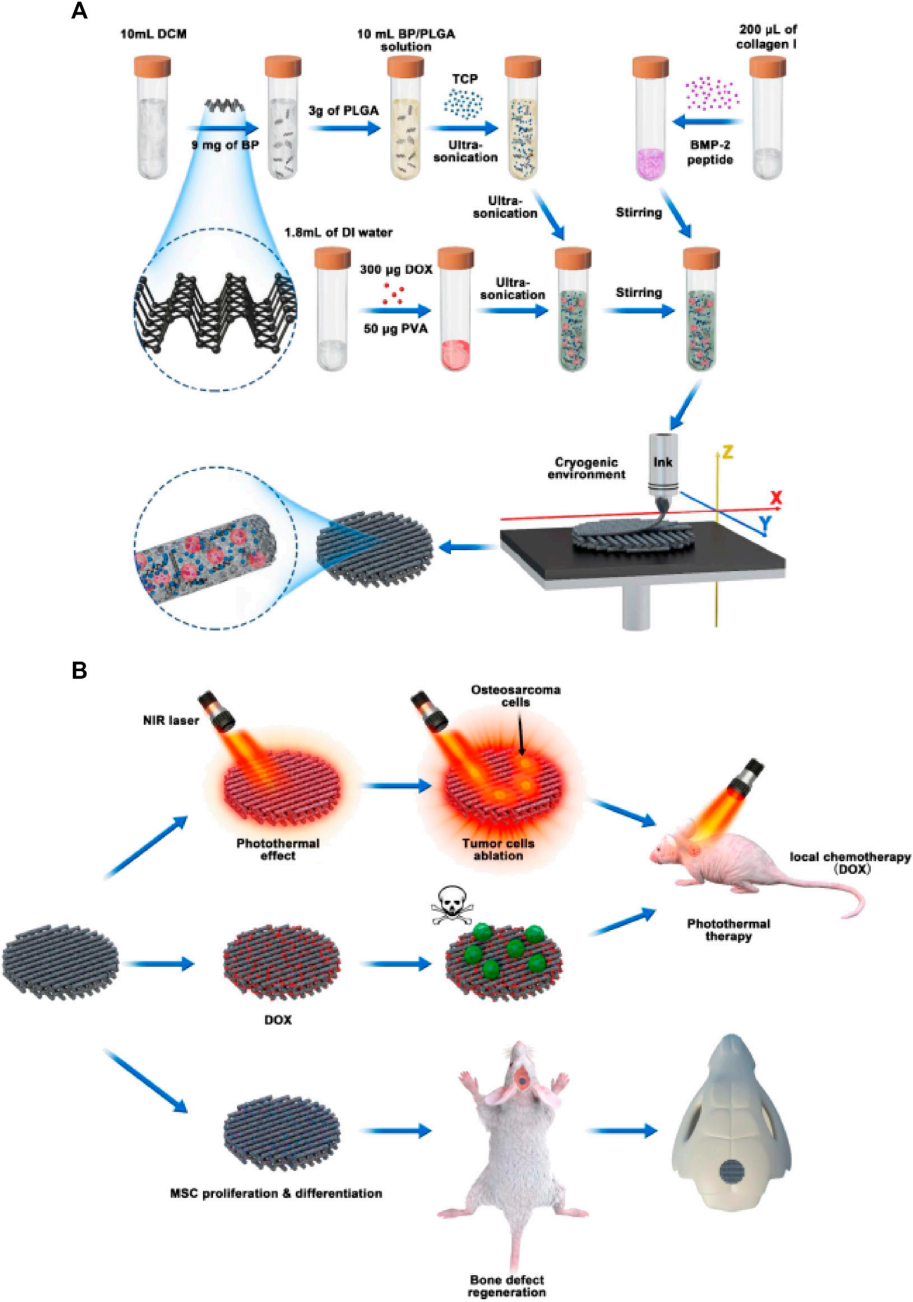
Schematic illustration of cryogenic 3D printing of multi-functional scaffolds and their multi-functions. **(A)** formulation of multi-delivery inks and 3D printing of multi-functional scaffolds; **(B)** tumor tissue ablation in nude mice through photo-thermal therapy and localized chemotherapy and regeneration of cranial bone defects of rats implanted with multi-functional scaffolds. Reproduced with permission from Wang C et al.

Using a 3D printing technique at low temperatures, Li et al. fabricated a scaffold composed of PLGA/β-TCP incorporating salvianolic acid B. Their results indicated that incorporating SB could promote the osseointegration of the obtained scaffolds by enhancing the effect of salvianolic acid B on angiogenesis and osteogenesis (Lin et al., 2019). However, the acidic product produced by the degradation of PLGA may cause an inflammation, which the incorporation of CaP materials can eliminate (Zhao et al., 2020b; Liang et al., 2020; Sokolova et al., 2020). In comparison to conventional chemotherapy, magnetic hyperthermia and photo-thermal therapy are attracting more attention because of their low invasive potential and high specificity. Li et al. fabricated a magnetic scaffold of PLGA, Fe_3_O_4_, and HA nanoparticles (Li et al., 2019b). It was found that the resulting scaffolds could raise the internal temperature to 47°C and induce substantial tumor cell apoptosis *in vitro*. In addition, the scaffolds showed an excellent osteogenic capacity to allow new bone to be regenerated in the defect site, with the result that bone volume/total was significantly higher than the untreated group (Li et al., 2019b). Wang et al. (Wang et al., 2020b)used black phosphorus nanosheets, DOX, and hydrophilic osteogenic peptides as photo-thermal agents, chemotherapeutic agents, and osteogenic factors, respectively, blended with 𝛽-TCP nanoparticle and PLGA for 3D printing to obtain drug delivery scaffolds for the treatment of bone tumor resection-induced defects (Figure 9). BDPTP scaffolds can cause tumor cell death *in vitro* and tumor clearance *in vivo*. The scaffold has a photo-thermal effect, and laser radiation with a wavelength of 808 nm can raise the BDPTP scaffold temperature to 60°C within 10 min. The MG63 cells attached to the scaffold were nearly eliminated after 1 day of culturing, the tumor disappeared after 4 days, and the tumor recurrence rate *in vivo* was low by combining chemotherapy and photo-thermal treatment. The *in vivo* experiments showed that the scaffold promoted new bone growth through its osteoinductive and osteogenic inductive effects (Wang et al., 2020b). Therefore, as a multi-functional platform, BDP-PT scaffolds can promote apoptosis and bone tissue regeneration in bone cancer, thereby meeting the clinical need of treating bone cancer.

Bone regeneration after resection of tumor tissue still faces a vast clinical obstacle in the therapy of bone defects, the therapeutic approaches that promote bone regeneration while being antimetastatic are of great interest. s. Hu et al. prepared drug delivery scaffolds by encapsulating BPQDs in PLGA nanoparticles and mixing them with wood/silk hydrogels to achieve adequate mechanical strength, bone formation, and tumor treatment (Hu et al., 2022). The drug delivery scaffolds further potently enhanced osteogenesis *in vivo* by efficiently enhancing bone mesenchymal stem cells’ growth, differentiation, and migration (Hu et al., 2022). More important, the BPQDs in the drug delivery scaffolds could suppress osteoclast differentiation and exert photo-thermal activity on spinal tumor metastasis (Hu et al., 2022). Significant inhibition of the growth of human osteosarcoma cells *in vitro* and reduction of tumor progression *in vivo* was also observed with the nanohydroxyapatite/collagen scaffolds filled with DOX-PLGA nanoparticles (Rong et al., 2016). These researches offer potential evidence for the possible treatment application in clinics for bone regeneration and bone metastasis ablation.

**FIGURE 10:**
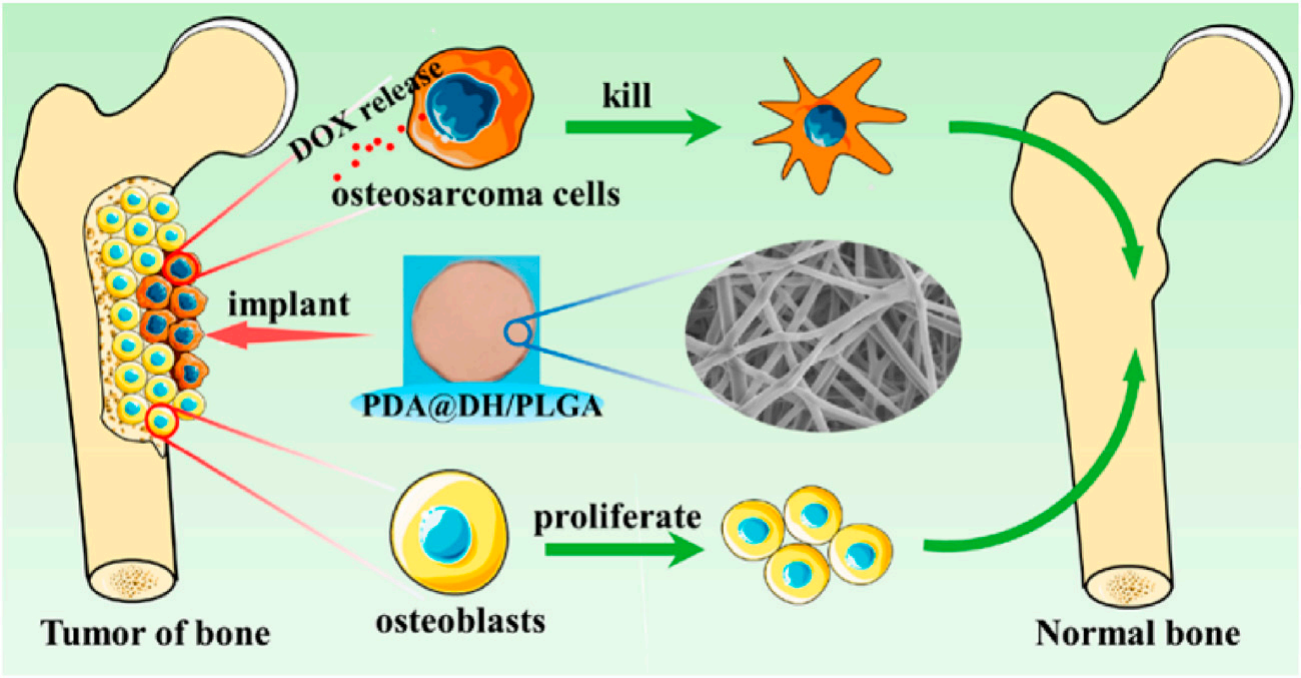
Schematic illustration of polydopamine on doxorubicin-loaded lamellar hydroxyapatite/PLGA composite fibers for inhibiting bone tumor recurrence and enhancing bone regeneration. Reproduced with permission from Lu Y et al. Copyright ^©^ 2021, American Chemical Society.

To overcome the difficulties of tumor reoccurrence and extensive bone defect, Long et al. designed an innovative multi-functional PLGA/Mg scaffold for the comprehensive postoperative management of osteosarcoma (Long et al., 2021), which resulted in the total inhibition of tumor reoccurrence when exposed to near-infrared laser radiation as well as the successful repair of bone defects *in vivo* (Long et al., 2021). In addition, the PLGA/Mg scaffold loaded with β-tricalcium phosphate (β-TCP) also exhibited both osteogenic and angiogenic capabilities, which had a synergetic effect in promoting the new bone generation and strengthening the quality of newly regenerated bone (Lai et al., 2019). The innovative PLGA/Mg scaffolds exhibited superior performance in suppressing postoperative osteosarcoma recurrence and regenerating bone, offering a potential clinically relevant approach for treating osteosarcoma. Lu (Lu et al., 2021) developed a composite scaffold coated with polydopamine, consisting of lamellar hydroxyapatite loaded with DOX and PLGA, to inhibit tumors and repair bones (Figure 10). The scaffold significantly inhibited tumor cell growth, followed by enhanced osteoblast adhesion and proliferation. Improved bone growth around the scaffold was also demonstrated *in vivo* (Lu et al., 2021). The dual-function scaffold holds significant potential for the therapy of bone tumors.

## 4 Conclusion

Overall, with several studies demonstrating their efficacy in improving survival in animal models, the progress of PLGA-based drug delivery systems in effectively inhibiting bone tumors and promoting bone tissue regeneration is very promising. However, there are still challenges associated with this technology. Most PLGA-based drug delivery systems do not achieve zero-level release, resulting in uneven local drug levels during treatment, and the accurately controlled distribution of the drug in time and space continues to be a significant challenge, making long-term tumor suppression difficult. An increase in drug load may benefit tumor treatment, while high concentrations of chemotherapeutic agents may cause local tissue toxicity that may be detrimental to bone reconstruction. Optimizing these systems and improving their targeting and delivery efficiency will be the subject of further studies. To overcome this significant hurdle, more detailed studies and extensive research are needed to develop new strategies to optimize drug release kinetics through interdisciplinary collaboration among experts in clinical medicine, materials science, and nanotechnology. Clinical trials will also be needed to evaluate the safety and effectiveness of these systems on human patients with bone tumors.
